# Celastrol-based nanomedicine promotes corneal allograft survival

**DOI:** 10.1186/s12951-021-01079-w

**Published:** 2021-10-26

**Authors:** Zhanrong Li, Ruixing Liu, Zhihua Guo, Dandan Chu, Lei Zhu, Junjie Zhang, Xintao Shuai, Jingguo Li

**Affiliations:** 1 Henan Eye Hospital, Henan Provincial People’s Hospital, People’s Hospital of Zhengzhou University, 450003 Zhengzhou, China; 2 PCFM Lab of Ministry of Education, School of Materials Science and Engineering, Sun Yat-Sen University, 510275 Guangzhou, China

**Keywords:** Corneal allograft rejection, Celastrol, Nanomedicine, Corneal penetration, M1 macrophages, TLR4

## Abstract

Effectively promoting corneal allograft survival remains a challenge in corneal transplantation. The emerging therapeutic agents with high pharmacological activities and their appropriate administration routes provide attractive solutions. In the present study, a celastrol-loaded positive nanomedicine (CPNM) was developed to enhance corneal penetration and to promote corneal allograft survival. The in vitro, in vivo and ex vivo results demonstrated the good performance of CPNM prolonging the retention time on ocular surface and opening the tight junction in cornea, which resulted in enhanced corneal permeability of celastrol. Both in vitro and in vivo results demonstrated that celastrol inhibited the recruitment of M1 macrophage and the expression of TLR4 in corneal allografts through the TLR4/MyD88/NF-κB pathway, thereby significantly decreasing secretion of multiple pro-inflammatory cytokines to promote corneal allograft survival. This is the first celastrol-based topical instillation against corneal allograft rejection to provide treatment more potent than conventional eye drops for ocular anterior segment diseases.

**Supplementary Information:**

The online version contains supplementary material available at 10.1186/s12951-021-01079-w.

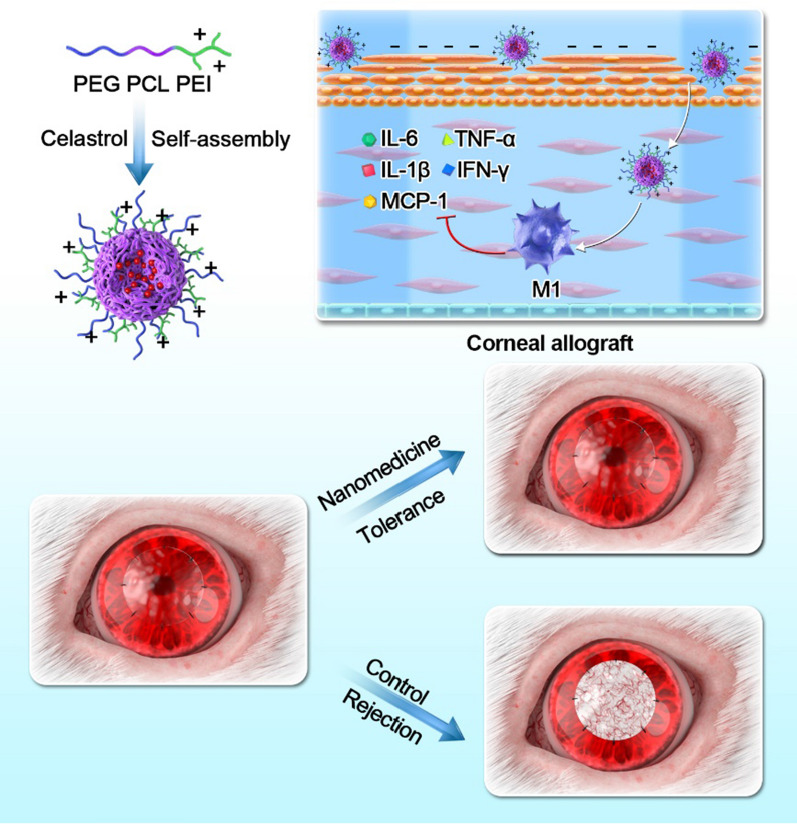

## Introduction

**Fig. 1:**
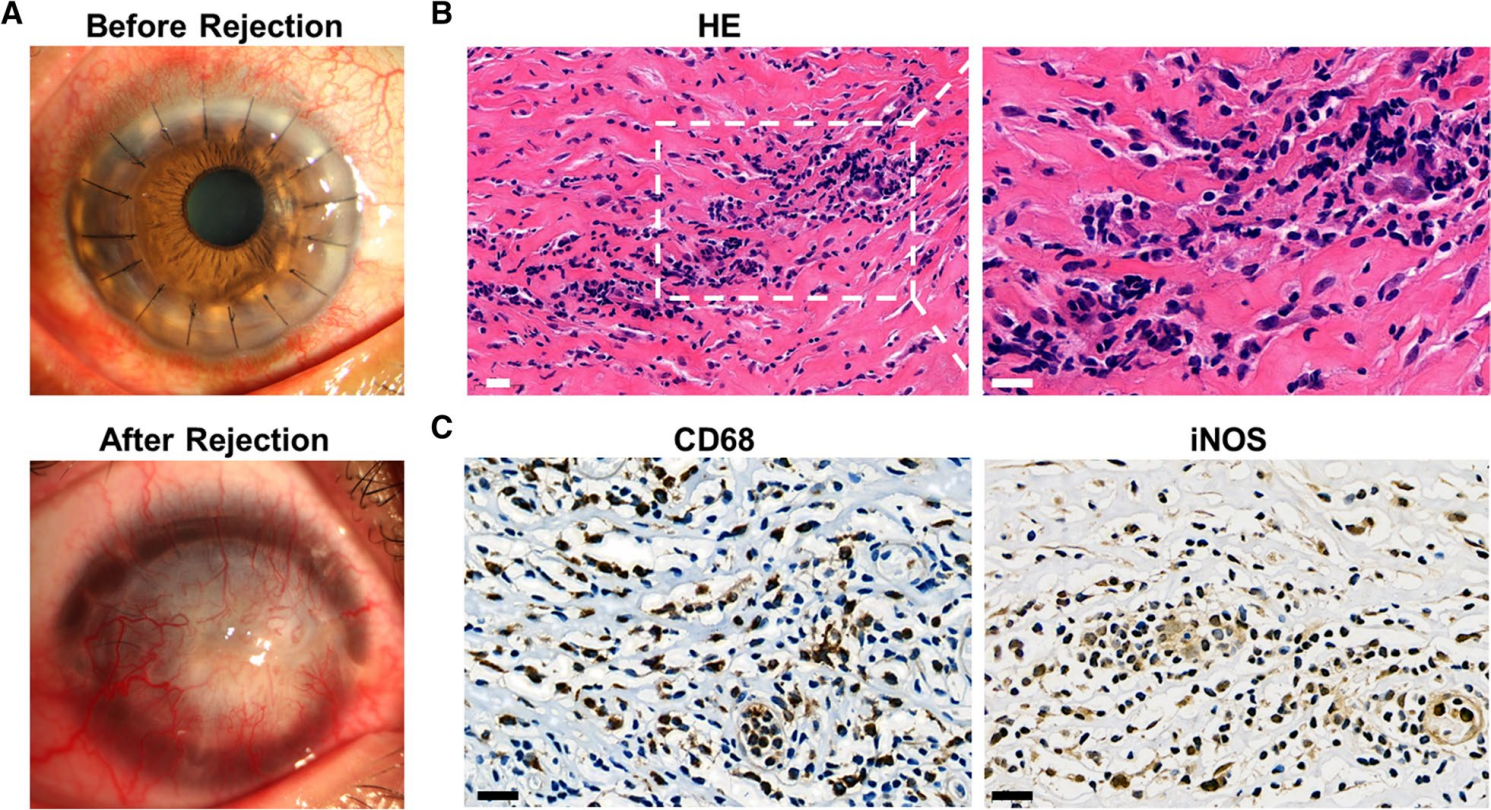
Corneal graft rejection. **A** The clear cornea before rejection and the opaque cornea and a large number of corneal neovascularization after rejection. **B** A mass of inflammatory cells infiltrated into the rejected corneal graft. **C** Most of the infiltrating inflammatory cells expressed CD68 and iNOS positive in the rejected cornea. Bar = 20 μm

Immune-mediated allograft rejection is the most common cause for corneal transplantation failure [1]. Corneal allograft rejection (CGR) is histologically characterized by the invasion of corneal neovascularization and infiltration of macrophages and CD4+ T cells [2]. We also found that a large number of CD68+ macrophages and iNOS+ cells in the rejected corneal tissue (Fig. 1). Activated macrophages are often classified as proinflammatory M1 macrophages or anti-inflammatory M2 macrophages [3]. Although the pathogenesis of CGR remains unclear, M1 macrophages have been reported to play a crucial role in the progress of CGR [4, 5] M1 macrophages induced by lipopolysaccharide (LPS) or IFN-γ express high level of inducible nitric oxide synthase (iNOS) [2]. M1 macrophages produce TNF-α, IL-1, IL-6 and IL-12, which are potent proinflammatory cytokines inducing inflammation and tissue destruction [5]. The TLR4/MyD88/NF-κB pathway was activated in the progress of kidney transplantation rejection [6]. However,whether there was an activation of TLR4 in CGR has not been validated yet.

Topical or systemic administration of corticosteroids and other immunosuppressive agents is considered efficient in improving corneal allograft survival. However, serious side effects including cataract, glaucoma, nephrotoxicity, hypertension and hepatotoxicity usually occur due to the high does or long-term medication [7, 8]. Therefore, there exists an urgent need to find new and more effective immunosuppressive agents. Celastrol (Cel), known as tripterine, is a pentacyclic-triterpene derived from the Trypterigium wilfordii Hook F called Thunder of God Vine. To date, numerous studies have demonstrated that celastrol has various biological activities including anti-inflammation, anti-angiogenesis, anti-tumor and anti-obesity [9–13]. It has been used for treating inflammatory and auto-immune disorders such as rheumatoid arthritis and Crohn’s disease [14, 15 ]. In our previous study, celastrol showed potent anti-corneal neovascularization and macrophage inhibition effects [16, 17]. Although celastrol is known to be a potent immunosuppressive and anti-inflammatory agent, the mechanism how celastrol alters innate immune response has not been explored thus far. The present study aimed at exploring the effects of celastrol on CGR and the possible mechanisms involved.

**Scheme 1:**
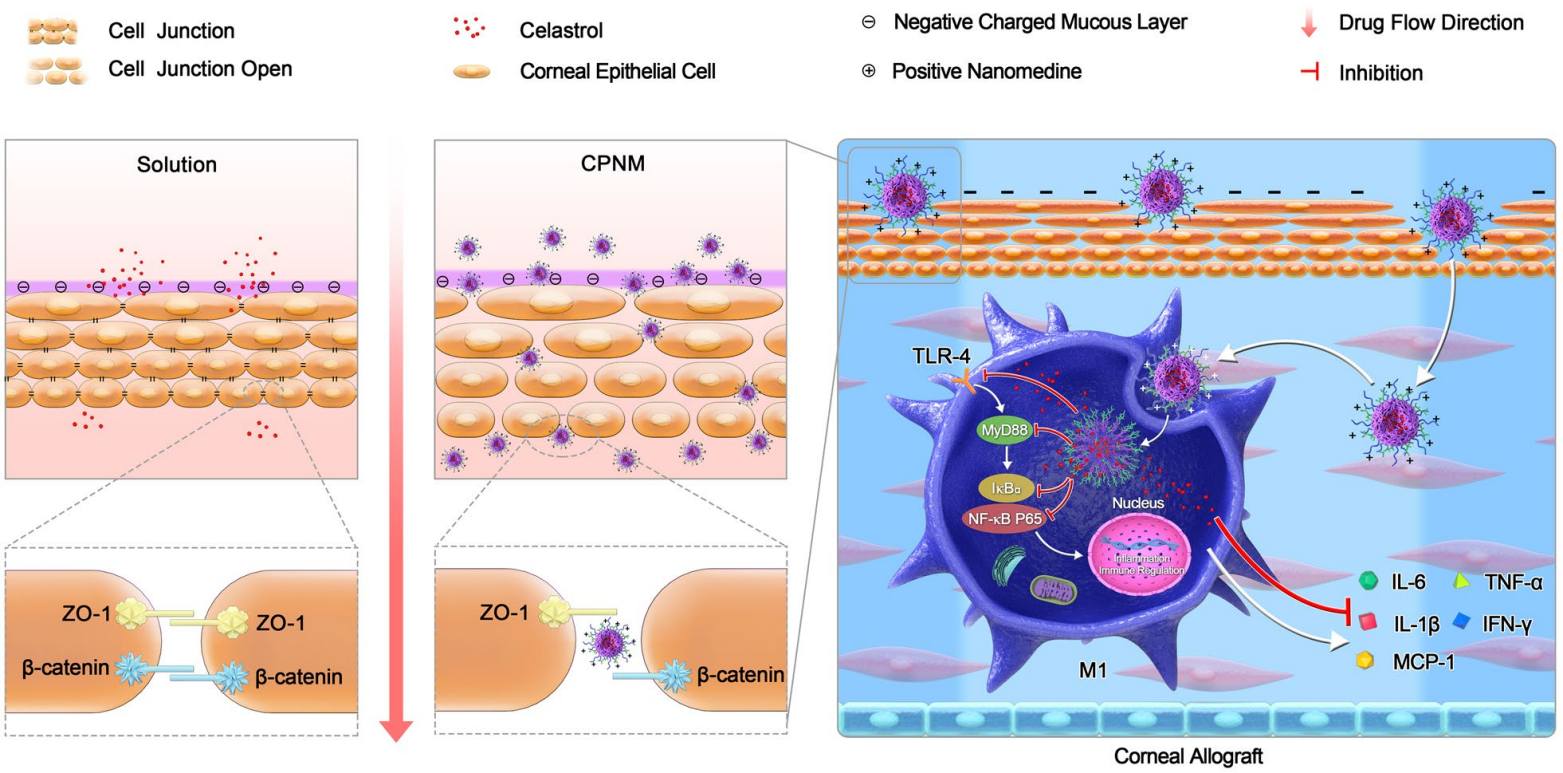
Schematic of celastrol-based nanomedicine enhance corneal penetration and promote corneal allograft survival

Despite its potential bioactivity, further therapeutic application of celastrol is affected by its poor water solubility. We recently developed celastrol-loaded nanomedicine (CNM) to overcome the poor water solubility impeding in vivo application, which boosted the therapeutic efficacy of celastrol [10, 16, 17]. Herein, we developed a celastrol-loaded positive nanomedicine (CPNM) to further prolong the retention time on ocular surface and to increase the corneal permeability of drug, which was expected to promote the survival corneal allografts (Scheme 1). The effect of CPNM on macrophages through TLR4/MyD88/NF-κB pathway was investigated.

## 2 Materials and methods

### Preparation and characterization of CPNM

Triblock copolymer poly(ethylene glycol)-poly(*ε*-caprolactone)-*g*-polyethyleneimine (PEG-PCL-g-PEI, PCI) was synthesized as previously described [18]. Celastrol (from the Shanghai Institute of Materia Medica, Chinese Academy of Sciences, Shanghai, China) was loaded into the PCI. PCI and celastrol were co-dissolved in methanol/acetonitrile (v/v = 1:1) and then added dropwise to PBS under ultrasonic agitation. The organic solvents were removed by vacuum distillation. Finally, CPNM was prepared after the solution was washed and filtered. Blank positive nanomedicine (PNM), blank nanomedicine (NM) and FDA (fluorescein diacetate)-loaded nanomedicine (FPNM) were also prepared following the same procedure as the above.

The hydrodynamic sizes and zeta potentials of PNM were determined by dynamic light scattering (DLS) using a Zeta Sizer Nano 90 (Malvern Instrument, UK). The morphology of PNM was investigated by transmission electron microscopy (TEM) using a Hitachi model H-7650 TEM. The celastrol loading content and in vitro release behaviour were quantified using HPLC system (Alliance 2695, Waters, USA) as previously described [17]. The ocular distribution and the penetration behaviors of the fluorescent model drug were examined in mouse eyes on two-photon laser scanning microscopy (Carl Zeiss Meditec AG, Jena, Germany).

### Immunocytochemistry analysis of β-catenin expression

Immunofluorescence was performed 1 h and 12 h after single topical PNM and NM dropping into the eye of rabbit. The concentration of PNM was the same as that of CPNM in vivo. Following removal of the eyeball, the fresh cornea was harvested and embedded in tissue-Tek optimum cutting temperature (O.C.T) compound. Corneal sections were cut transversely along the temporal-nasal axis of the eyeball. The frozen section (8-µm thick) were washed. Tissue specimens were fixed and then washed. Sections were treated with 0.3% Triton X-100 for 30 min, and then washed. Corneal sections were treated with bovine serum albumin and then incubated with rabbit polyclonal antibody against β-catenin. Immunoreactivity was evaluated using FITC-labeled antibodies, and cells were counterstained with 4′-6-diamidino-2-phenylindole (DAPI). Samples were examined using fluorescence microscopy.

### Immunofluorescence

Human corneal epithelial cells (HCECs) were seeded onto coverslips that had been placed into the wells of a six-well plate (5 × 10^5^ cells/well). Immunofluorescence staining was performed 1 h after PNM treatment to examine the localization and semi-quantification of ZO-1-positive cells in the HCECs. Next, the cells were washed, fixed and then blocked with 5% BSA. The cells were then incubated with ZO-1. Immunoreactivity was evaluated using FITC-labeled antibodies, and the cells were counterstained with DAPI. Samples were examined using fluorescence microscopy.

### In vivo rabbit ocular irritation test

As previously described, a single-dose eye stimulation test was conducted in three healthy New Zealand white rabbits [19]. The right eye received 40 µL of CPNM, while the left eye was used as a control. The cornea, conjunctiva, iris, and anterior chamber were inspected for inflammation or toxic reaction. Furthermore, both eyes were stained with fluorescein and examined under slit lamp at 1, 24, 48, and 72 h after exposure. The eye irritation levels were scored using the Draize test. After irritation tests, the rabbits were sacrificed by air injection. The eyeball was excised and fixed in 4% paraformaldehyde and stained with hematoxylin eosin (n = 3).

### Animals

The eight-week-old female SD (Sprague-Dawley) and Wistar rats are with an average weight of 180–200 g. The animals were supplied by Beijing Vital River Laboratory Animal Technology Co. Ltd. The 8-week-old male C57BL/10ScNJNju (wild type) and C57BL/10JNju (TLR4−/−) mice are with an average weight of 20 g. All animals fed in barrier system test room had free access to a standard diet and drinking water. The New Zealand Rabbits weigh between 2 and 2.5 kg. All the experimental process was in accordance with the Henan Provincial People’s Hospital guidelines for the welfare of experimental animals.

### Corneal transplantation and postoperative treatment

Corneal transplantation was performed as previously described [4]. Briefly, excising a circular 3.0-mm central area of cornea made the recipient graft bed in SD rat. A central 3.5-mm diameter corneal button was excised from a donor using a trephine in Wistar rat. The donor cornea was transplanted to the recipient bed with eight interrupted 10-0 nylon sutures followed by the application of antibiotic ointment. Only the right eyes of SD rats were used in the experiments. All transplanted rats received tobramycin eye ointment postoperatively were randomly distributed into two groups (8 rats of each group). Group 1 (control) rats received PNM dispersion drops three times a day, while Group 2 (CPNM) rats received a topical instillation (20 µL) three times a day. The sutures were not removed till the end of observation period (40 d). All the treatments described above were administered to the grafted eyes from the day of transplantation until euthanasia.

### Clinical evaluation of corneal transplantation rats

All grafts were evaluated via a slit-lamp microscope digital system every other day. Graft transparency, edema, and neovascularization were used to evaluate the corneal grafts as previously described [4, 7]. Three parameters have five grades (0–4 scores) respectively (Additional file 1: Table S1). Grafts were considered to be rejected when the total scores are equal or greater than six. Rats with complications, including infection, cataract, or hemorrhage were eliminated from the study.

### Histology and immunohistochemistry of cornea

The rats were sacrificed using 10% chloral hydrate on the 8th d after transplantation, and the eyes from two groups were excised and fixed in 4% paraformaldehyde for histologic analysis. And then the removed corneal grafts were cut into 5-µm thick sections and stained with hematoxylin and eosin (HE). The number of inflammatory cells was counted in histological images of corneal grafts. To further investigate the mechanisms of CPNM effects in CGR, macrophage infiltration was evaluated in the transplanted corneas. The formalin-fixed corneal sections deparaffinized with ethanol and antigen were retrieved in retrieval solution. Anti-CD68 antibody and iNOS antibody were used as primary antibody. Immunohistochemical staining was carried out. Then, the numbers of infiltrated macrophages (CD68+) and iNOS+ cells in the corneal stroma were counted in five randomly selected fields under light microscope. For double-label immunofluorescence staining, corneal section (8 μm) were fixed by 4% paraformaldehyde. CD68 and TLR4 primary antibodies were added and incubated for 1 h, and the specimens were washed thoroughly with PBS and then were incubated with secondary antibody (Alexa Fluor 488 and Alexa Fluor 594) at room temperature for 1 h. Subsequently, corneal sections were stained with Hoechst 33,342 solution. The sections were observed by confocal fluorescence microscopy.

### Cell culture and treatment

The mouse macrophages (Raw264.7) were purchased from the Type Culture Collection of the Chinese Academy of Sciences (Shanghai, China), and were cultured in Dulbecco’s modified Eagle’s medium (DMEM) containing 10% fetal bovine serum, 100 U/mL penicillin, 100 µg/mL streptomycin, as well as 4.5 g/mL glucose at 37 °C in 5% CO_2_. The Raw264.7 cells were seeded on glass coverslips in DMEM added with 10 % FBS incubate for 24 h. The cells were respectively pre-treated with blank nanomedicine or CPNM for 12 h, and then activated with LPS for another 12 h. Thioglycolic acid-elicited macrophages were collected from the peritoneal cavity of mouse according to a previously described method [20]. Briefly, the cells were isolated from peritoneal lavage fluid samples collected on 4 days after intraperitoneal injection of 2 mL of thioglycolic acid. The cells were dispensed and cultured overnight in DMEM with 10% FBS and maintained at 37 °C and 5% CO_2_. The cells were respectively pre-treated with PNM or CPNM for 12 h.

### Immunofluorescence

Cluster of differentiation F4/80 (ab6640, abcom, USA) was used as a primary antibody. Fluorescein isothiocyanate (FITC)-conjugated goat anti-Rat immunoglobulin G (H+L) (SA00003-11, Proteintech, USA) was used as the secondary antibody. The nuclei were stained with Hoechst 33342 (Sigma-Aldrich, USA). To further investigate the inhibitory effect of the CPNM on macrophages, TLR4/MyD88/NF-kB P65 pathway was evaluated by immunofluorescence. The cells with and without CPNM pre-treatment were activated by LPS, fixed and then blocked with Goat Serum. Next, the cells were incubated with primary antibodies against TLR-4, NFκB-p65, P-NFκB-p65, IκBα, P-IκBα, MyD88, TRIF, exposed to Alexa Fluor® 488, and stained with Hoechst 33,342 for cell nuclei detection. Images were captured under a confocal laser scanning microscopy.

### Flow cytometry

The mouse macrophages were harvested, washed, and incubated with FITC-dextran and Alexa Fluor® 647. The macrophages were washed twice with PBS after incubation and the percentage of intracellular FITC-dextran was confirmed by flow cytometry. In addition, it was also used to identify the purification of mouse macrophages from the peritoneal cavity by flow cytometry. The expressions of CD80, iNOS, CD206 and Arg1 in macrophages were analyzed after LPS treatment. The effect of CPNM on TLR4 expression in macrophages was analyze by flow cytometry.

### Protein array analysis of cytokines in culture supernatant of peritoneal macrophages and corneal grafts

The amounts of secreted cytokines were compared between wide type and TLR4−/− peritoneal macrophages with CPNM treatment using C-Series Mouse Cytokine Antibody Array C1 for 22 mouse cytokines as directed by the manufacturer. Briefly, culture supernatants were collected from peritoneal macrophages induced by LPS for 12 h after pretreatment with CPNM, ST2825 (MyD88 inhibitor) and BAY11 (NF-kB inhibitor). The expression levels of 22 different cytokines were assessed using a Mouse Cytokine Array kit. Chemiluminescence signals produced in proportion to the number of cytokines were detected using FluorChemQ. Meanwhile, cytokines were compared in the corneal grafts between two groups on the 8th day after surgery with the C-Series Rat Cytokine Antibody Array C1. The signal intensity of the spots was quantified using image J software.

### Reverse transcription-polymerase chain reaction (RT-PCR)

The gene specific primers used are shown at Additional file 1: Table S2. Gene expression analysis for cytokine factors were performed on the 8th d after transplantation in control and CPNM groups. Total RNA was isolated from corneal tissue using Trizol. Total RNA was extracted with chloroform and proazamine. Then, RNA was reversely transcribed with a QuantiTect RT-PCR kit, and complementary DNA was subsequently amplified using TaqPCR SuperMix. Results are expressed as fold over untreated control.

### Western Blot (WB) analysis

Macrophages (wild type and TLR4−/−) were pretreated with PNM or CPNM for 12 h in advance and activated by LPS to determine TLR4/NF-κB pathway protein expression. Proteins were extracted using RIPA buffer, and the protein concentrations were measured using a BCA protein assay kit. An equal amount of each protein sample was separated by SDS-PAGE and electro transferred onto polyvinylidene difluoride membranes. After blocking, the membranes were probed with antibody TLR-4, NFκB-p65, P-NFκB-p65, IκBα, P-IκBα, MyD88, TRIF, β-actin and β-Tubulin. Subsequently, the membranes were incubated with IgG HRP secondary antibody, followed by imaging with a chemiluminescent substrate. The signal was detected by FluorChemQ and analyzed using Image J.

### Statistical analysis

Data was presented as mean ± SD and analyzed via one-way ANONA or Student *t*-test. *P* < 0.05 was considered to be statistically significant. Long-term survival proportion of corneal allograft survival was analyzed with Kaplan-Meier survival curves and log-rank test.

## Results

### Preparation and characterization of CPNM

**Fig. 2:**
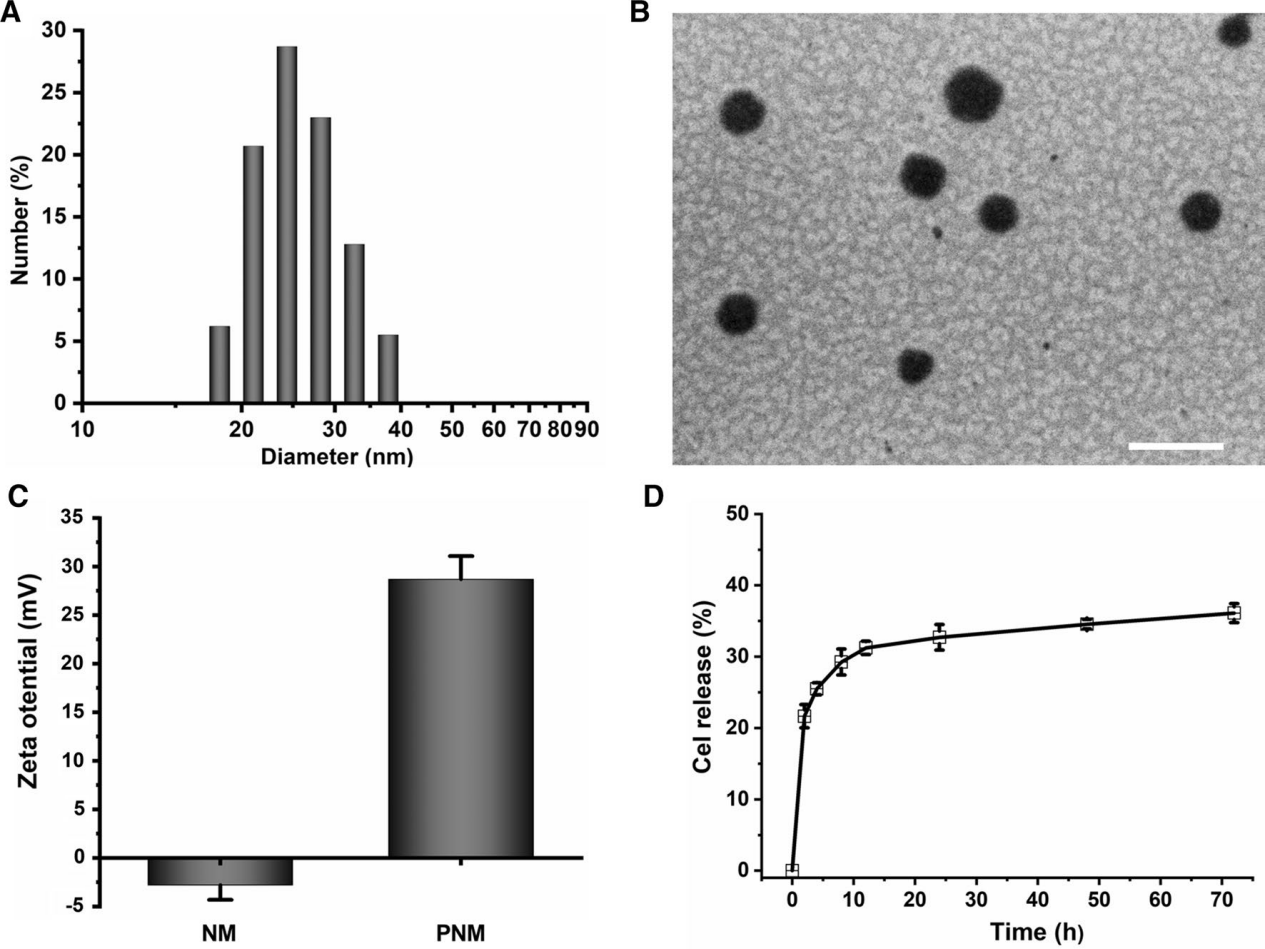
The physicochemical properties of PNMs. **A** The particle size of PNM; **B** Transmission electron microscopy (TEM) images of PNMs stained with uranyl acetate. The scale bars were 50 nm; **C** The zeta potential of PNMs and NMs, Mean ± SD, n = 3; **D** In vitro release profiles of celastrol from CPNMs in PBS (pH 7.4) at 37 °C

The triblock copolymer PCI was synthesized via multi-step reactions, and CPNM were prepared as reported [18]. As measured by dynamic light scattering (DLS), the hydrodynamic diameter and zeta potential of PNMs were 24.94 ± 5.42 nm and +28.70 ± 2.37 mV, respectively (Fig. 2A, C, and Additional file 1: Figure S1), indicating that the nanomedicine has a small particle size and a positive zeta potential. The morphology of PNMs appeared to be spherical in shape (Fig. 2B). The celastrol loading content of CPNM was determined to be 6.58%, and celastrol in CPNM displayed a typical biphasic release profile in vitro release (Fig. 2D), which was similar to the previous reports [17, 18].

### Enhanced corneal penetration of PNM

**Fig. 3:**
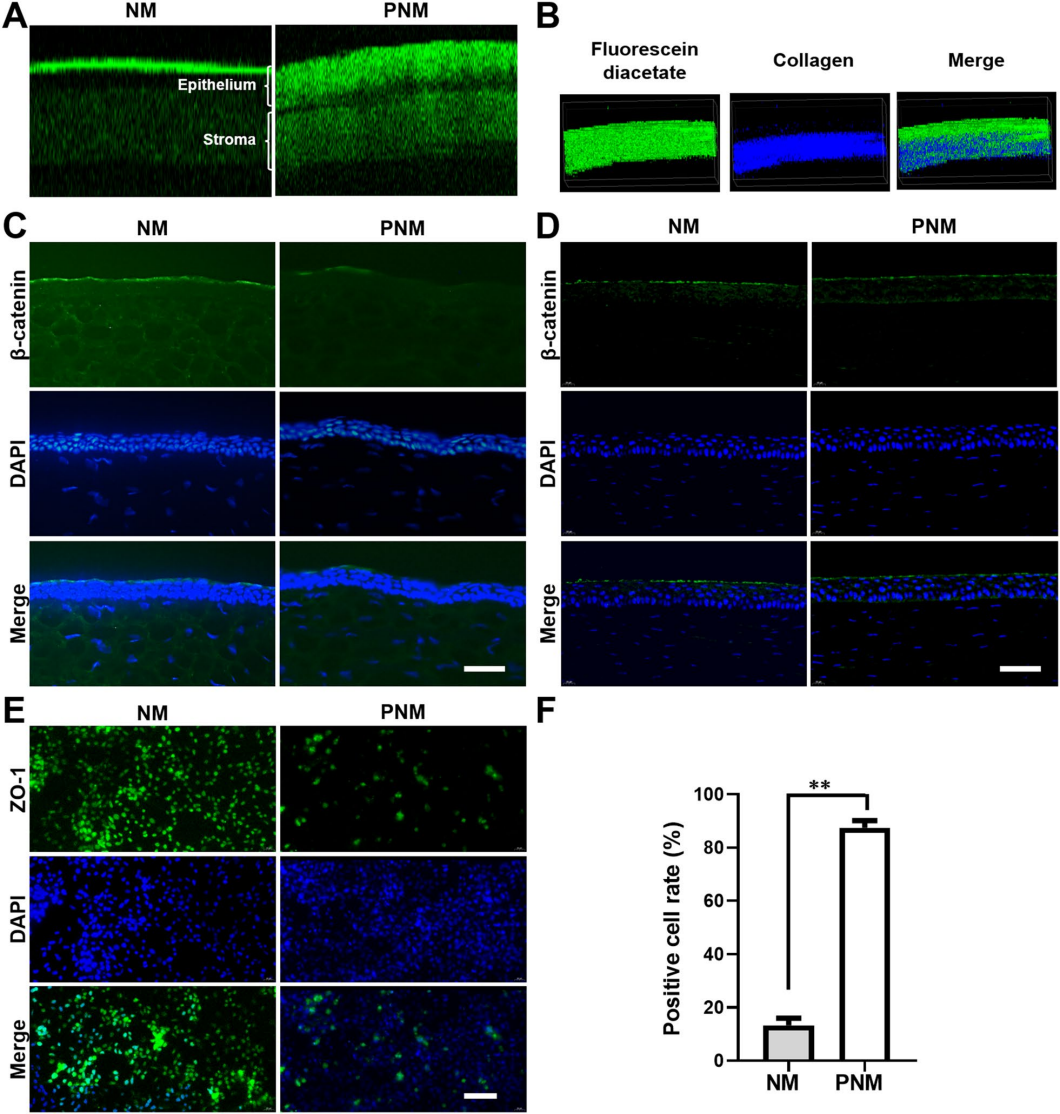
The corneal penetration behaviors of nanomedicines. **A** Representative in vivo two-photon microscopy 2D images of corneal cross-sections at 1 h after administration of NM and PNM in C57Bl/6 mice; **B** Representative in vivo two-photon microscopy 3D images of corneal after administration PNM in C57Bl/6 mice; **C** Representative micrographs of corneal sections obtained from each group stained with DAPI (blue fluorescence) and anti-β-catenin (green) at 1 h and **D** 12 h after topical application. DAPI staining, blue. Merge, colocalization image of β-catenin and DAPI; **E** Confocal laser scanning microscopy images of HCECs cell layers immunofluorescent stained with DAPI (blue fluorescence) and ZO-1 (green fluorescence) after incubation with CNM and CPNM for 1 h and **F** the statistical analysis results. DAPI staining, blue. Merge, colocalization image of ZO-1 and DAPI. Bar = 50 μm

Corneal penetration performance of PNM was investigated through in vivo 3D imaging, in vitro immunofluorescence staining and ex vivo immunohistochemistry. Using two-photon microscopy to observe the cross-sections of transparent cornea tissue in real time, we estimated the in vivo corneal penetration of PNM. In our previous study, the PCI polymeric micelle was observed to penetrate cornea effectively in a time-dependent manner [18]. However, the mechanism has not been fully explored yet. Hence, a detailed in-depth study was carried to gain insight into the penetrating mechanism. As shown in Fig. 3A, the strong fluorescence signal was determined both in the epithelial layers and stromal layers at 1 h after administrating FPNM eye drops (Fig. 3A, B). In contrast, as for the PEG-PCL micelle (nanomedicine, NM) which had a similar particle size (24.70 ± 6.68 nm) and a neutral zeta potential (− 2.78 ± 1.52 mV) (Fig. 2C and Additional file 1: Figure S1), the fluorescent signal was mostly limited to the epidermis of corneal epithelium in addition to a relatively weak signal in stromal layers. Furthermore, the results of 2D and ortho images of corneal indicated that the positively charged nanomedicine was mostly located in the extracellular region of corneal epithelial cells (Additional file 1: Figure S2).

To evaluate the corneal permeability mechanism of the positively charged nanomedicine in the cornea tissues, the ex vivo immunohistochemistry assay of β-catenin was carried out. As shown in Fig. 3C and D, in the nanomedicine group, β-catenin was mainly present at the superficial layer of the corneal epithelium with a continuous distribution. However, after treatment with the positive nanomedicine, fluorescence distribution became discontinuous and the fluorescence intensity of corneal epithelial declined at 1 h (Fig. 3C), surprisingly, the distribution of β-catenin resumed continuity in corneal epithelium at 12 h (Fig. 3D). It indicated that the function of cell junctions was temporarily altered.

To investigate the penetrating mechanisms of PNM, immunofluorescence staining of ZO-1, an expressed protein indicating the tight junction, was employed to assess the tight junction-opening capability of the positive micelle in vitro [21]. As shown in Fig. 3E, in the nanomedicine treatment group, cells retained their tight junctions due to the integrity of corneal epithelial barriers. However, after treatment with PNM, the ZO-1 expression was gradually decreased. The fluorescence intensity decreased by 6.6-fold (87.45% vs. 13.31 %, *P* < 0.0001) (Fig. 3E), implying that PNM could open the tight junctions. These results revealed that the positively charged nanomedicine could open the tight junction in cornea to enhance drug penetration.

### In vivo rabbit ocular irritation test

**Fig. 4:**
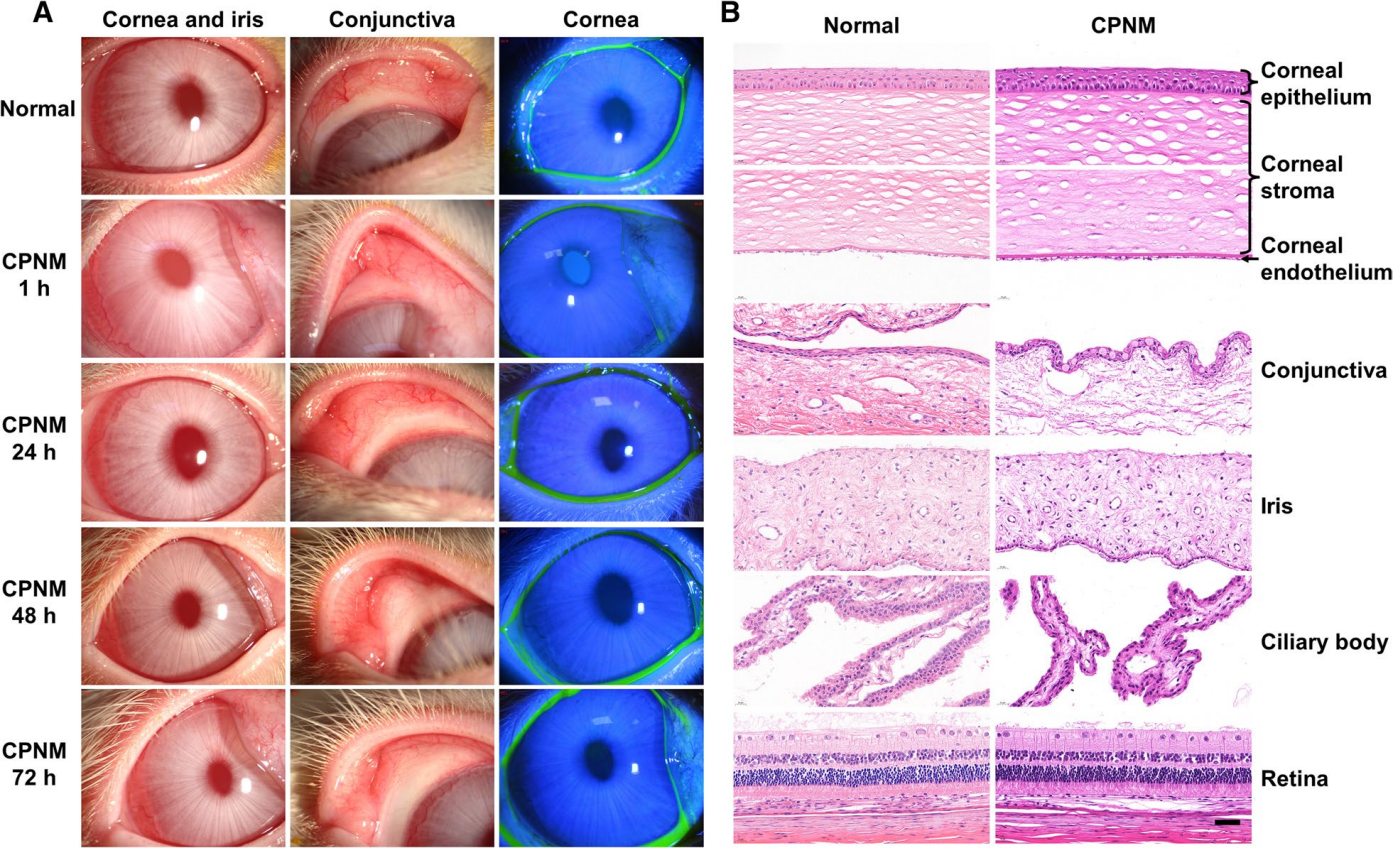
The toxicity and eye-irritation testing of CPNM. **A** Photo documentations and fluorescein staining by Slit-lamp including cornea, iris and conjunctiva in normal and CPNM groups; **B** HE staining in 72 h. Scale bars: 50 μm

CPNM were nonirritant and could be tolerated by the rabbit eye. Cross sections from rabbits’ eye after application of CPNM together with a control section showed that both tissue structure and integrity were unaffected (Fig. 4).

### CPNM treatment prolonged corneal allograft survival

**Fig. 5:**
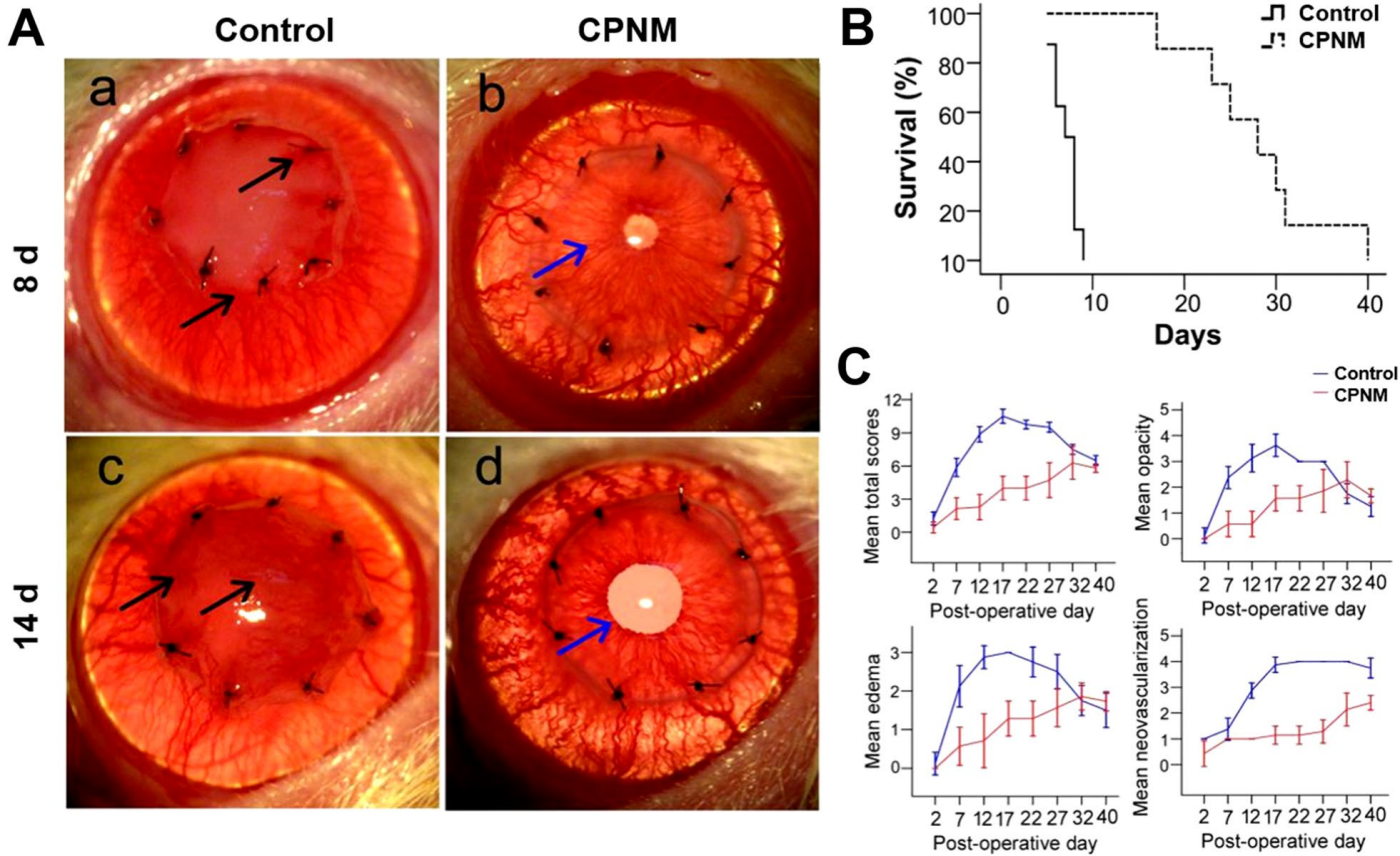
CPNM prolonged the corneal allograft survival. **A** Clinical manifestations (magnification, ×250) of the anterior segment on postoperative 8 days and 14 days. Representative photographs of the corneal allografts which were treated with or without CPNM at post-operative 8 days and 14 days. **B** Survival curves of rat corneal grafts for two groups. The average survival time of corneal graft in the control group was about 7.3 ± 0.6 days, while that in the CPNM group was 29.1 days ± 2.4 days. **C** Corneal transparency, edema, corneal neovascularization and total scores of each group were compared. Within about 27 days after surgery, scores in the CPNM group were significantly lower than those in the control group, with statistically significant differences

We performed allogeneic corneal transplantation using Wistar rats as donors and SD rats as recipients. The effects of CPNM treatment on the corneal allografts were assessed for 40 days after transplantation (Fig. 5). Almost all the grafted corneas showed pronounced rejection, showing obvious corneal opacity, edema and neovascularization at 8 days after surgery in control group. On the contrary, the corneal grafts were clear in the CPNM group (Fig. 5A). The survival curves are summarized in Fig. 5B. All corneal grafts in the control group were rejected within 10 days, showing a mean survival time of 7.25 ± 0.59 days (n = 8). In contrast, all corneal grafts in the CPNM group could survive up to 40 days, i.e., till the end of observation, showing a mean survival time of 29.13 ± 2.36 days (n = 8). These results clearly showed that the CPNM treatment significantly prolonged the survival time of corneal allograft as compared with the blank micelle treatment (*P* < 0.01). The average rejection score, opacity, edema, and neovascularization were compared between the two groups for each time point, and significant differences existed three weeks after surgery (*P* < 0.05) (Fig. 5C).

### CPNM suppressed M1 macrophage recruitment and TLR4 expression in corneal allografts

**Fig. 6:**
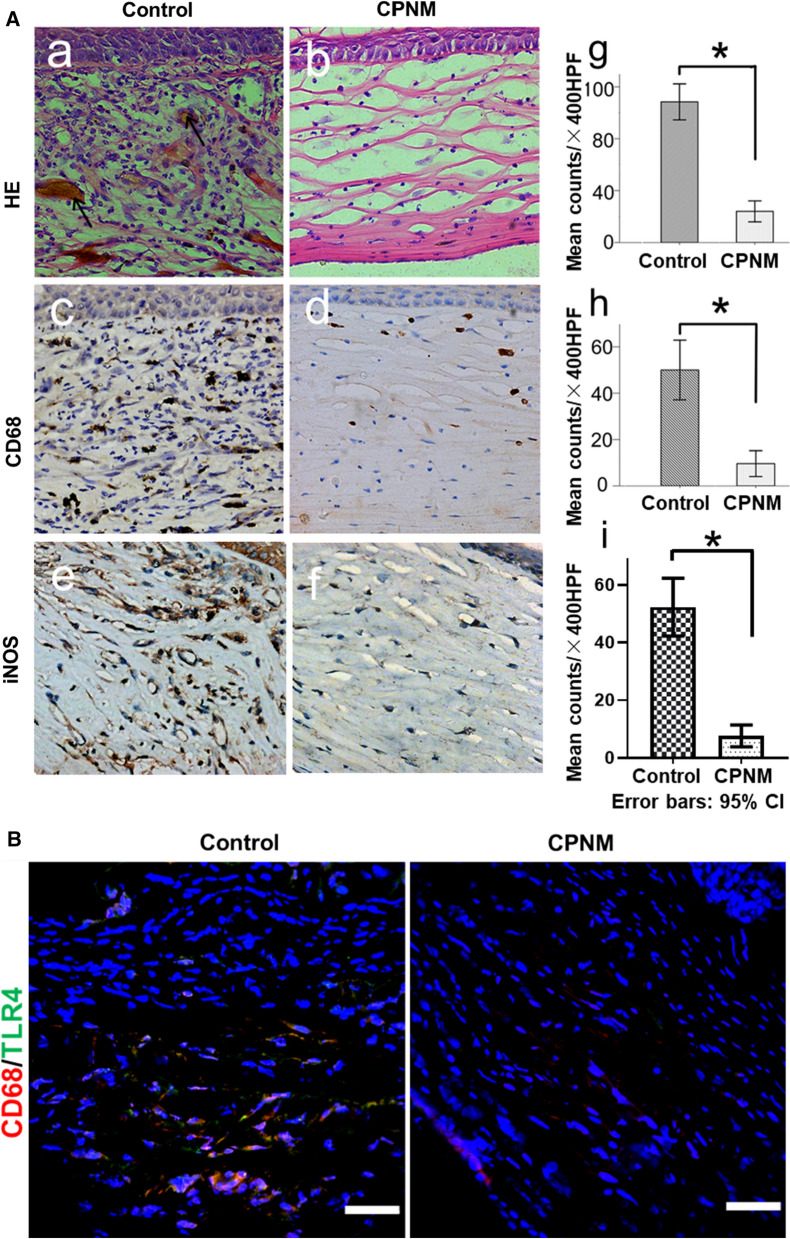
CPNM inhibited macrophages recruitment and TLR4 expression in corneal allografts. **A** HE and immunohistochemical staining of corneal grafts on 8 days after surgery (magnification, ×200). **A** (**a**, **b**) HE stains showing the infiltration of inflammatory cells in the allografts of CGR rats in two groups. Immunohistochemical staining of CD68+ (**c**, **d**) and iNOS+ (**e**, **f**) cells. Greater numbers of neovascularization, CD68+ macrophages and iNOS+ expression (stained in brown) were observed in the blank control group than in the CPNM treated group. The number of inflammatory cells (**g**), macrophages (**h**) and iNOS+ expression cells (**i**) infiltration was statistically significant. *P < 0.05. **B** Co-labeling in macrophages (CD68 for red and TLR4 for green) from different groups. Scale bars: 50 μm

To further investigate the mechanism of CPNM-mediated beneficial effects on CGR, macrophages infiltration was evaluated in the corneal allografts and the histopathology of corneas were analyzed using HE and immunohistochemical staining for the two groups (Fig. 6). As shown in the HE staining, the allografts in the control group exhibited substantial infiltration of inflammatory cells as well as edema at the postoperative 8 days. By contrast, the CPNM treatment group exhibited fewer inflammatory cells as well as much less edema. To further investigate the mechanism of celastrol-mediated effects on CGR, macrophage infiltration in the transplanted corneas was evaluated. Immunohistochemical staining of the corneal grafts showed decrease in numbers of CD68+ and iNOS+ cells which are closely related to the immune responses of macrophages. Immunofluorescence double-staining showed substantial CD68/TLR4 co-labeling in the infiltrating macrophages in the corneal allografts of the control group. The CPNM group exhibited a dramatically reduced proportion of proinflammatory macrophages (TLR4+ or iNOS+ cells) at 8 days post-operation (Fig. 6A, B).

### CPNM inhibited expressions of proinflammatory cytokine and chemokine in corneal allografts

**Fig. 7:**
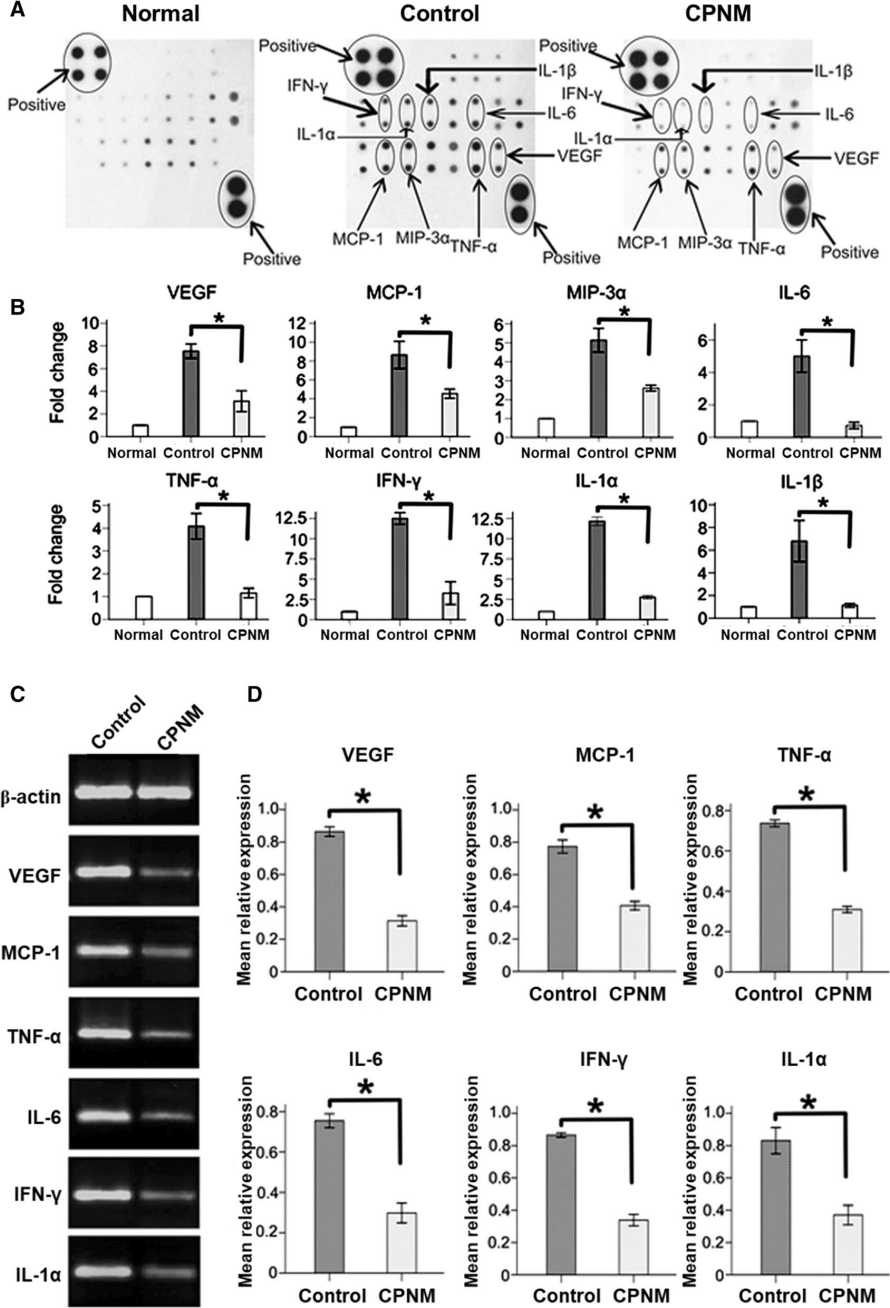
CPNM reduced the protein and mRNA expressions of pro-inflammatory cytokines and chemokines in corneal allografts at 8 days after transplantation. **A** The expressions of VEGF, MCP-1, MIP-3α, IL-6, TNF-α, IFN-γ, IL-1α and IL-1β from different groups and **B** statistical analysis of relative protein levels of cytokines. Mean optic densities of protein were calculated by normalizing to controls. **C** The expressions of VEGF, MCP-1, TNF-α, IL-6, IFN-γ, and IL-1α from different groups and **D** the statistical analysis of relative mRNA levels of cytokines. *P < 0.05

Macrophages activated by the TLR4 pathway secreted a wide variety of pro-inflammatory cytokines and chemokines such as IL-1, IL-6, TNF-α, IFN-γ and MCP-1 [22]. MCP-1 and macrophage inflammatory protein-3α (MIP-3α) are two major chemokines in recruitment of macrophages or monocytes [23]. We explored the effects of CPNM on the expressions of these chemokines in grafts at 8 days after transplantation. As shown in Fig. 7A, B, the protein expression levels of IL-1α, IL-1β, IL-6, TNF-α, IFN-γ, MCP-1 and MIP-3α were lowered in the corneal allografts which were administrated with CPNM, as compared with the administration of blank nanomedicine. It is known that VEGF plays a key role in the development of corneal neovascularization in addition to promoting inflammation *via* increasing vascular leakage and promoting monocyte chemotaxis [16]. Excitingly, the expression of VEGF was suppressed in corneal allografts treated with CPNM. Quantification of the gene expression levels using RT-PCR to quantify the target gene expressions at mRNA level obtained consistent results. As shown in Fig. 7C, D, the corneal allografts treated with CPNM had much lower expression levels of IL-1α, IL-6, TNF-α, IFN-γ, MCP-1 and VEGF than the control corneas.

### CPNM downregulated TLR4 expression in M1 macrophages

**Fig. 8:**
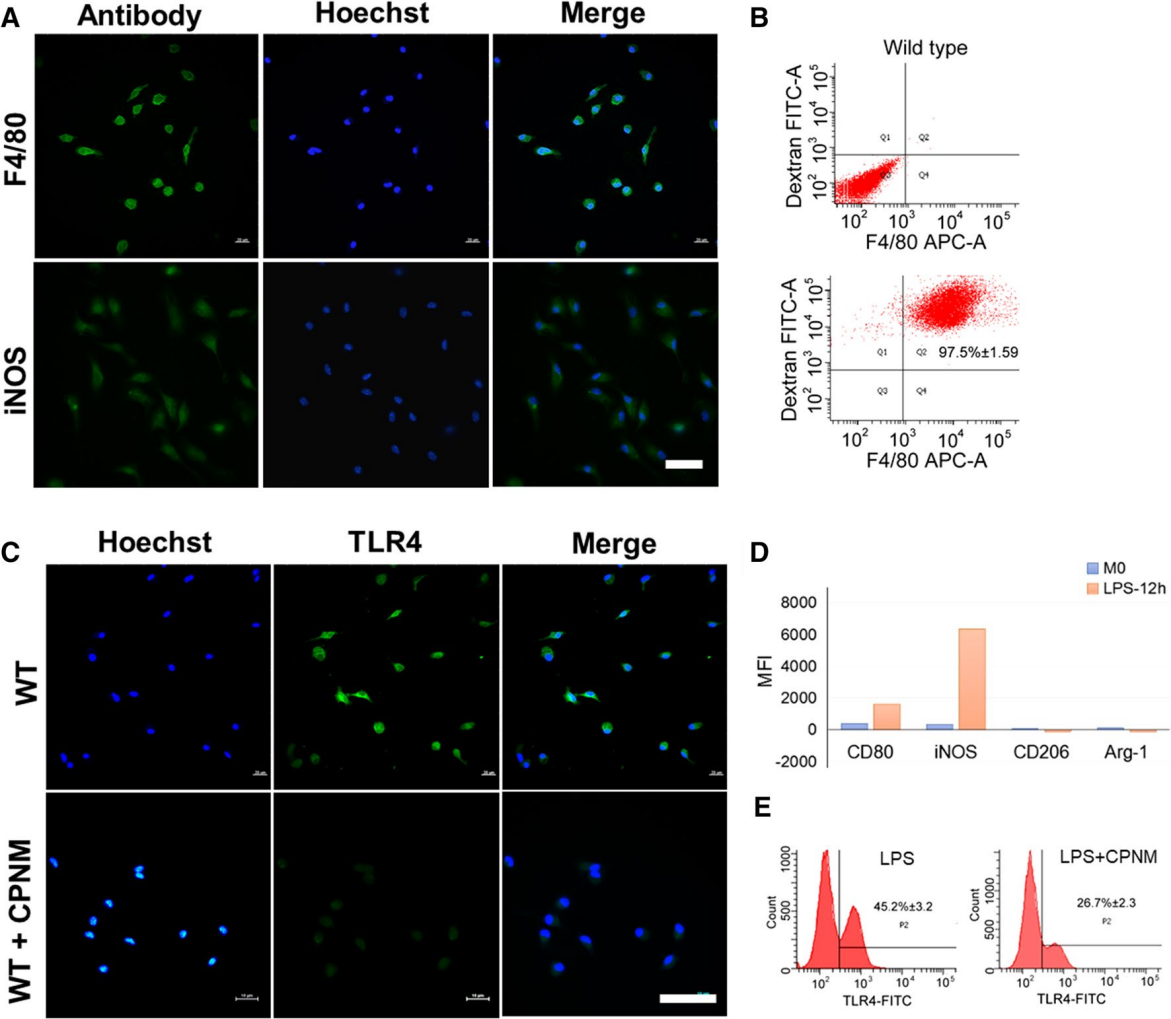
Celastrol downregulated the expression of TLR4 in M1 macrophages. **A** Peritoneal cells elicited by thioglycolic acid highly express F4/80 and iNOS in wild type mouse. **B** The ratio of F4/80 and dextran co-labeling cell is 97.5% + 1.59%. **C** The expression of TLR4 reduced notably in CPNM group in wild type M1 macrophages. **D** The MFI of CD80, iNOS, CD206 and Arg-1 is respectively 1617, 6371, -107 and -64. **E** Celastrol reduced the ratio of TLR4 from 45.2% ± 3.2 to 26.7% ± 2.3% in macrophages activated by LPS

To investigate how CPNM regulates macrophages, we further examined the effect of CPNM on the expression of TLR4 in macrophages. It was found that peritoneal cells elicited by thioglycolic acid highly expressed F4/80, iNOS and TLR4 in wild type mice (Fig. 8A, C). The ratio of cell co-labeling F4/80 and dextran reached 97.5 % (Fig. 8B), suggesting that macrophages elicited by thioglycolic acid were M1-type polarized. As shown in Fig. 8C, CPNM down-regulated the expression of TLR4 in wild type M1 macrophages. To further verify the effect of celastrol on TLR4 expression in M1 macrophages, we evaluated the effect of celastrol on LPS-induced macrophages. The results showed that LPS stimulation elevated the expressions of CD80 and iNOS in RAW264.7 cells, whereas the expressions of CD206 and Arg-1 did not change significantly after LPS stimulation. The median fluorescence intensities (MFI) of CD80, iNOS, CD206 and Arg-1 were 1617, 6371, -107 and -64, respectively (Fig. 8D), which suggested that LPS-induced macrophages exhibited M1 phenotype. Notably, the increased TLR4 expression in macrophages due to LPS stimulation was reduced to 26.7% from 45.2% via CPNM pretreatment (Fig. 8E).

### CPNM suppressed expressions of proinflammatory cytokines and chemokines in LPS-activated macrophages

**Fig. 9:**
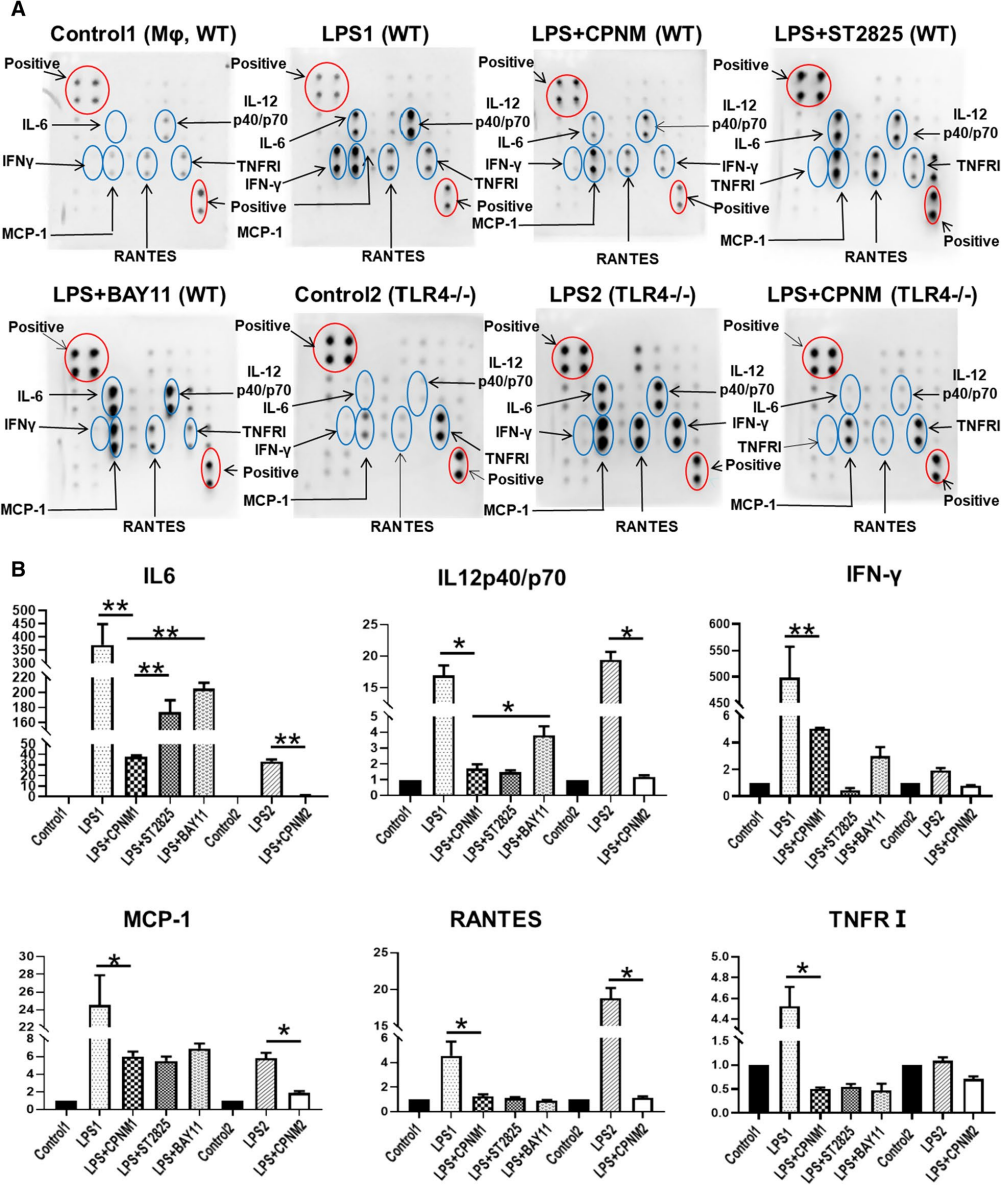
CPNM attenuated cytokines secretion in M1 macrophages. **A** Detection of cytokine expression profiles in supernatants from LPS-inducing peritoneal Mϕ. Supernatants from TLR4−/− Mϕ and wild-type Mϕ pretreated with CPNM, ST2825 and BAY11 for 24 h and normal medium (control) were assessed using a mouse cytokine antibody array. The downregulation of IL-6, IL12p40/p70, IFN-γ, MCP-1, RANTES and TNFR I was shown in the supernatants from LPS-inducing wild-type Mϕ after CPNM pretreatment. There was no significant difference in the expression of IFN-γ and TNFR I in LPS-inducing TLR4−/− Mϕ with CPNM. **B** Statistical analysis of relative protein levels of cytokines. Mean densities of protein were calculated by normalizing to controls. The data indicates the mean ± SD (n = 3), *P < 0.05 and **P < 0.01

The effects of CPNM on the activation of macrophages were assessed using the peritoneal macrophages elicited by thioglycolic acid in wild type and TLR4−/− mouse. The macrophages were pretreated with blank nanomedicine and CPNM (1 µg/mL) for 12 h and with ST2825 (10 µmol/L) and BAY11 (5 µmol/L) for 24 h, respectively. Then, the macrophages were activated with LPS (1 µg/mL) for 12 h. In comparison with the wild type macrophages only activated with LPS, the wild type macrophages receiving CPNM treatment showed notably decreased expression levels of IL-6, IL-12p40/p70, IFN-γ, MCP-1, RANTES and tumor necrosis factor receptors I (TNFR I). Moreover, the TLR4−/− macrophages activated with LPS showed much lower expression levels of IL-6, MCP-1, IFN-γ and TNFR I than the wild type macrophages. However, the expression levels of IL-12p40/p70 and RANTES seemed to be upregulated in the TLR4−/− macrophages. In comparison with the TLR4−/− macrophages only treated with LPS, the TLR4−/− macrophages receiving CPNM treatment showed significantly decreased expression levels of IL-6, IL-12p40/p70, MCP-1 and RANTES. The IFN-γ and TNFR I expressions in the TLR4−/− macrophages were not obviously changed upon the CPNM treatment (Fig. 9). These results revealed that the celastrol treatment mediated with nanocarrier could effectively inhibit the macrophage activation and corresponding cytokines secretion.

### CPNM inhibited TLR4/MyD88/NF-κB pathway in macrophages

**Fig. 10:**
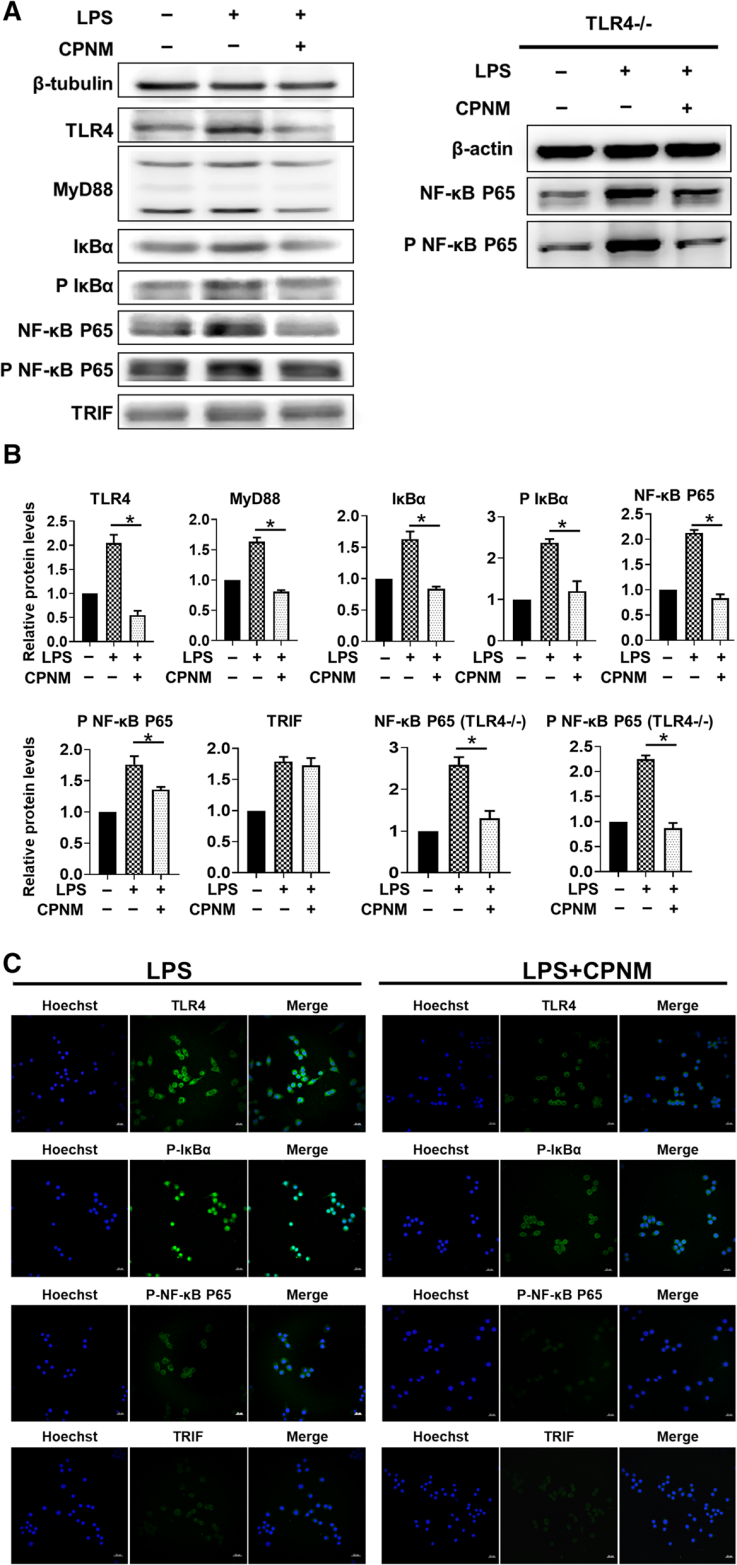
CPNM inhibited TLR4/MyD88/NF-κB P65 signal pathway in M1 macrophages. **A** The expressions and **B** statistical analysis of relative protein levels of TLR4, MyD88, IκBα, NF-κB P65 and TRIF in M1 Mϕ detected by WB after pretreating with CPNM for 12 h at the indicated concentrations, *P < 0.05 and **P < 0.01. **C** Immunofluorescence showed that CPNM inhibited TLR4/NF-κB P65 pathway in M1 macrophage. The expressions of TLR4, p IκBα and p NF-κB P65 significantly decreased in CPNM group. There was no significant difference in the expression of TRIF

To further evaluate the effect of celastrol treatment on the TLR4/MyD88/NF-κB signaling pathway, both immunofluorescence and WB assay were performed to detect the signaling pathway in the Raw264.7 and TLR4−/− macrophages. After CPNM pretreatment, macrophages were exposed to LPS in order to assess the effect on cellular signaling *via* NF-κB and TRIF, respectively. Compared to the control cells, the cells activated with LPS showed more expressions of TLR4, MyD88, IκBα, pIκBα, NF-κB P65 and pNF-κB P65, all of which could be reduced upon the CPNM treatment. However, there was no significant difference in the TRIF expression between different groups. In addition, CPNM treatment also downregulated the expressions of NF-κB P65 and pNF-κB P65 in TLR4−/− macrophages induced by LPS (Fig. 10A, B). Similar to the WB results, immunofluorescent staining showed that the TLR4, pIκBα and pNF-κB P65 protein levels were significantly increased in the LPS activation group but decreased in the CPNM treatment group, as compared with their expressions in the control group, while no obvious difference in the TRIF expression was observed between different groups (Fig. 10C). These results clearly showed that the celastrol treatment inhibited the M1 macrophages activation via the TLR4 and MyD88-dependent signaling rather than a TRIF signaling pathway.

## Discussion

CGR is a complex immune inflammatory reaction involving multiple inflammatory cells and proinflammatory mediators, and macrophages play a key role in the early acute CGR [25–27]. Macrophages display remarkable plastic phenotype and diverse functions [28]. The M1 macrophage is induced by toll-like receptor (TLR) ligands LPS, tumor necrosis factor alpha (TNF-α), and interferon gamma (IFN-γ). They secrete numerous pro-inflammatory cytokines like IL-1, IL-6, IL-12, IFN-γ, and TNF-α [29, 30]. M1 macrophages exert high antigen-presenting capacity [5]. On the other hand, M2 macrophages are polarized by cytokines such as IL-4 and IL-13 [31]. M2 macrophages secret cytokines like IL-10 and TGF-β to promote tissue repair via immune tolerance and tissue remodeling [32]. Thus far, reliable evidences have shown that the iNOS-positive proinflammatory (M1) macrophages were recruited in CGR. We have also observed a large number of M1 macrophages expressing TLR4 and iNOS in acute CGR. It shows that M1 macrophages mainly contribute to the pathogenesis of acute CGR [3, 4].

In the present study, we found that topical CPNM administration significantly prolonged corneal allograft survival, with reduced corneal neovascularity and corneal edema, inhibited macrophages infiltration, and lowered expressions of iNOS and TLR4 in corneal allograft. Moreover, CPNM treatment suppressed the expressions of IL-6, IFN-γ, TNF-α, MCP-1, IL-1α and VEGF at protein and mRNA levels in corneal allograft. However, we could not rule out the possibility that these cytokines might have derived not only from macrophages but also from lymphocytes or activated resident cells. Moreover, CPNM treatment reduced the proportion of TLR4 and the secretion of inflammatory cytokines and chemokines in macrophages activated by LPS. Therefore, we speculate that celastrol may prolong corneal graft survival through inhibiting M1 infiltration and down-regulating the secretion of inflammatory cytokines and chemokines. Hence, to ascertain the definitive role of celastrol and the relative roles of different macrophage subsets in allograft rejection and dysfunction, further studies would be necessary. A better understanding of celastrol biology and immunomodulatory mechanisms underlying macrophage differentiation and phenotypic maturation would provide novel therapeutic targets for preventing or treating allograft rejection in the cornea and other organs.

TLR4 is one of TLRs receptors which contribute to macrophage infiltration, and its signaling has an important regulatory role in stimulating the expressions of immune and inflammatory response-related genes [33]. It has been reported that TLR4 is initiated through two different pathways, the MyD88-dependent pathway and the MyD88-independent (TRIF) pathway in macrophages [34]. Our findings indicated that celastrol inhibited the expressions of TLR4, MyD88, IκBα, pIκBα, NF-κB P65 and pNF-κB P65 in macrophages induced with LPS but showed no effect on the TRIF expression. Meanwhile, celastrol suppressed the CD68+/TLR4+ macrophages infiltration in corneal allograft. These results further elucidated the anti-inflammatory mechanisms of celastrol. Therefore, celastrol might have inhibited macrophages *via* TLR4/MyD88/NF-κB pathway to decrease the secretion of inflammatory mediators and to act against acute CGR. This is a clear indication that targeting TLR4 may provide a promising intervention strategy for the prevention or treatment of CGR. Our study showed the therapeutic effects of celastrol in CGR and further supported that celastrol might be a promising option for the treatment of organ transplantation.

Celastrol shows effective anti-inflammatory activity. However, its poor water solubility limits its application [35]. In our previous study, we developed a polymeric micelle to improve the water solubility of celastrol [10,13,16,17]. However, the special biological barriers of the eye affect the corneal permeability of micellar drugs, resulting in a significant decline in the bioavailability of drugs [36, 37]. In this study, we developed a nanomedicine with a small particle size around 25 nm and a zeta potential about + 29 mV. The results demonstrated that the positively charged nanomedicine not only prolonged the ocular residence time via the electrostatic interactions between their positively charged groups and the negatively charged mucin on the ocular surface (Fig. 3A), but also, even more importantly, was able to open temporarily the intracellular junctions (Fig. 3C) [ 38, 39]. On this basis, the small size of CPNM allowed them to easily cross the corneal epithelium by paracellular route (Fig. 3B, D, Additional file 1: Figure S2) [10, 18]. Furthermore, the outstanding solubility of the PEGylated shells endowed CPNM with an excellent permeability to effectively cross the amphipathic cornea [40, 41]. Taken together, CPNM exhibited a significantly penetration behaviour through the cornea. In the experimental model, pharmacological treatment via topical instillation of CPNM in this work instead of subconjunctival injection of CNM in our previous study revealed a significantly prolonged corneal allograft survival, possibly due to the enhanced corneal penetration and targeted delivery to corneal graft which enhanced the pharmacological activity of celastrol. Our findings demonstrated that PNM might be a promising system for effective ophthalmic drug delivery.

In summary, we demonstrated the heavy infiltration of M1 macrophages, high expression of TLR4, iNOS and proinflammatory cytokines and chemokines in rat corneal allografts with acute rejection. A topical instillation of CPNM significantly prolonged the survival of corneal allograft and decreased the infiltration of M1 macrophages and expression of TLR4, proinflammatory cytokines and chemokines in rats. Celastrol may suppress infiltration of M1 macrophage and secretion of inflammatory cytokines through TLR4/MyD88/NF-κB pathway. Given its favorable corneal permeability, this well-defined positive nanomedicine may be a viable alternative to conventional eye drops for the treatment of the ocular anterior segment diseases.

## Supplementary Information

**Additional file 1. Figure S1.** The particle size of PNMs and NMs. **Figure S2.** Representative 2PH microscopic 2D (A) and ortho images (B) of corneal in C57BL/6 mice in vivo. **Figure S3.** The biocompatibility of CPNM. **Figure S4.** The cytotoxicity of PNM and NM. **Figure S5.** The expressions of TLR4, MyD88, IκB-α, P IκB-α, NF-κB P65 and P NF-κB P65 decreased in TLR4-/-Mϕ. **Figure S6.** Survival curves of rat corneal grafts in each group. **Table S1.** Clinical Scoring system for the corneal transplantation rejection. **Table S2.** Primer used in RT-PCR assay. **Table S3.** Template showing the location of cytokine antibodies spotted onto the rat cytokine array c1. **Table S4.** Template showing the location of cytokine antibodies spotted onto the rat cytokine array c1.

## Data Availability

All data and material are included in the article and its additional files.

## Abbreviations

CGR: Corneal allograft rejection
LPS: Lipopolysaccharide
iNOS: Inducible nitric oxide synthase
TNFR I: Tumor necrosis factor receptors I
TLR: Toll-like receptor
TNF-α: Tumor necrosis factor alpha
IFN-γ: Interferon gamma
Mϕ: Macrophages
DAPI: 4’-6-Diamidino-2-phenylindole
HCECs: Human corneal epithelial cells
MFI: Median fluorescence intensities
Cel: Celastrol
CPNM: Celastrol-loaded positive nanomedicine
CNM: Celastrol-loaded nanomedicine
