# Supplementary material for: Celastrol-based nanomedicine promotes corneal allograft survival
Source: J Nanobiotechnology. 2021 Oct 26;19:341. doi: 10.1186/s12951-021-01079-w (PMC8549351; doi:10.1186/s12951-021-01079-w)

*Additional Information*

**Celastrol-based Nanomedicine Promotes Corneal Allograft Survival**

Zhanrong Li ^a^, Ruixing Liu ^a^, Zhihua Guo ^a^, Dandan Chu ^a^, Lei Zhu ^a^,*, Junjie Zhang, Xintao Shuai ^b^,*, Jingguo Li ^a^,*

^a^ Henan Eye Hospital, Henan Provincial People’s Hospital, People’s Hospital of Zhengzhou University, Zhengzhou 450003, China.

^b^ PCFM Lab of Ministry of Education, School of Materials Science and Engineering, Sun Yat-Sen University, Guangzhou 510275, China.

* Corresponding author. Henan Eye Hospital, Henan Provincial People’s Hospital, People’s Hospital of Zhengzhou University, Zhengzhou 450003, China

E-mail address: lijingguo@zzu.edu.cn (J. Li), hnyks135@126.com (L. Zhu), shuaixt@mail.sysu.edu.cn (X. Shuai).


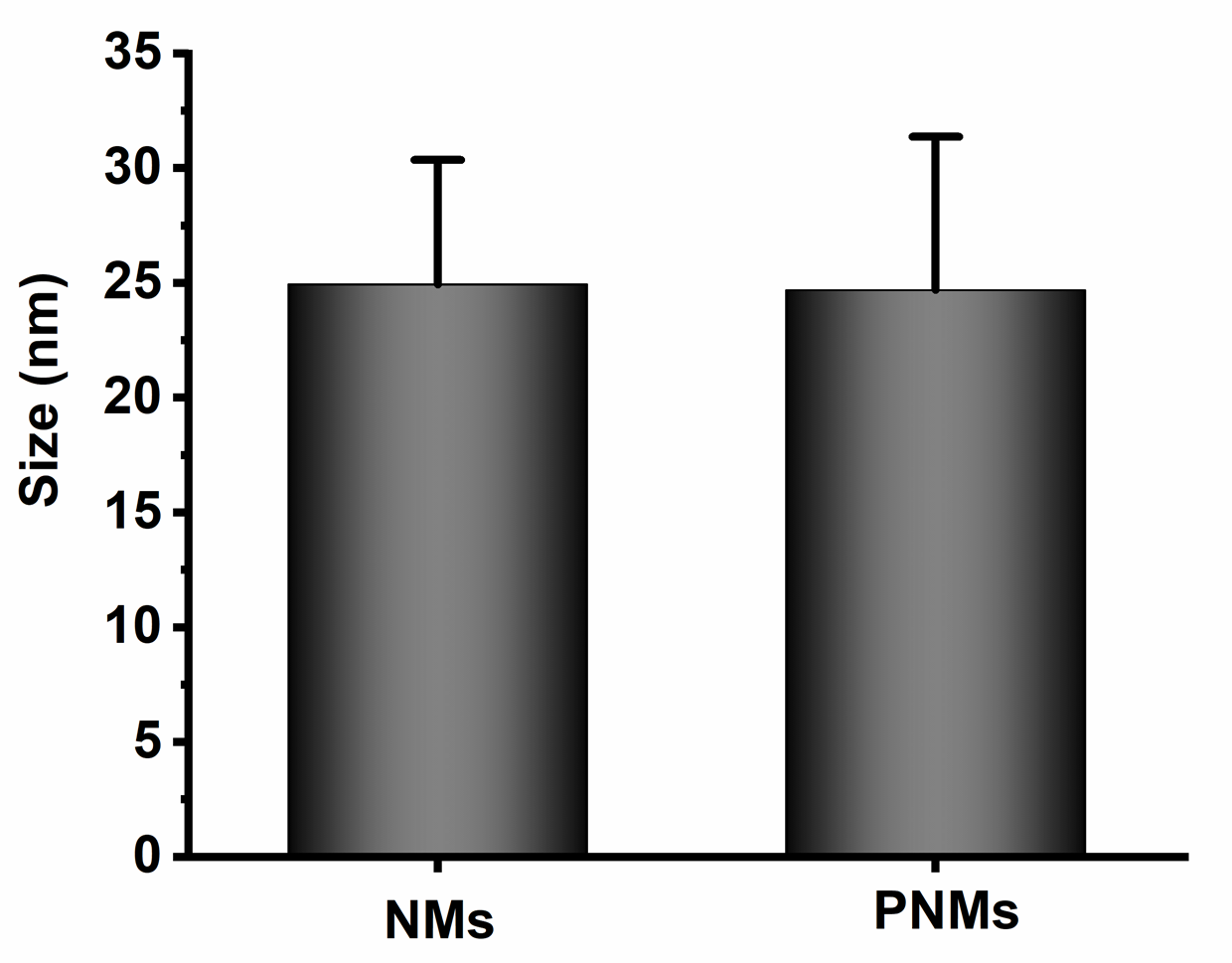


**Figure S1.** The particle size of PNMs and NMs, Mean ± SD, n = 3.


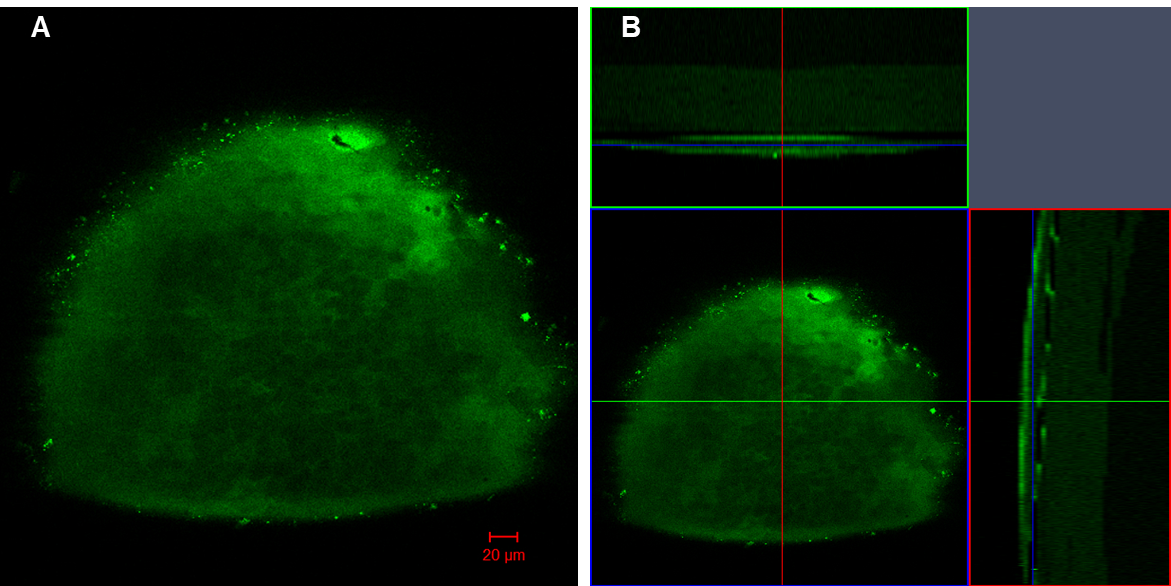


**Figure S2.** Representative 2PH microscopic 2D (A) and ortho images (B) of corneal in C57BL/6 mice in vivo.

**Figure S3.** The biocompatibility of CPNM.

**Figure S4.** The cytotoxicity of PNM and NM.


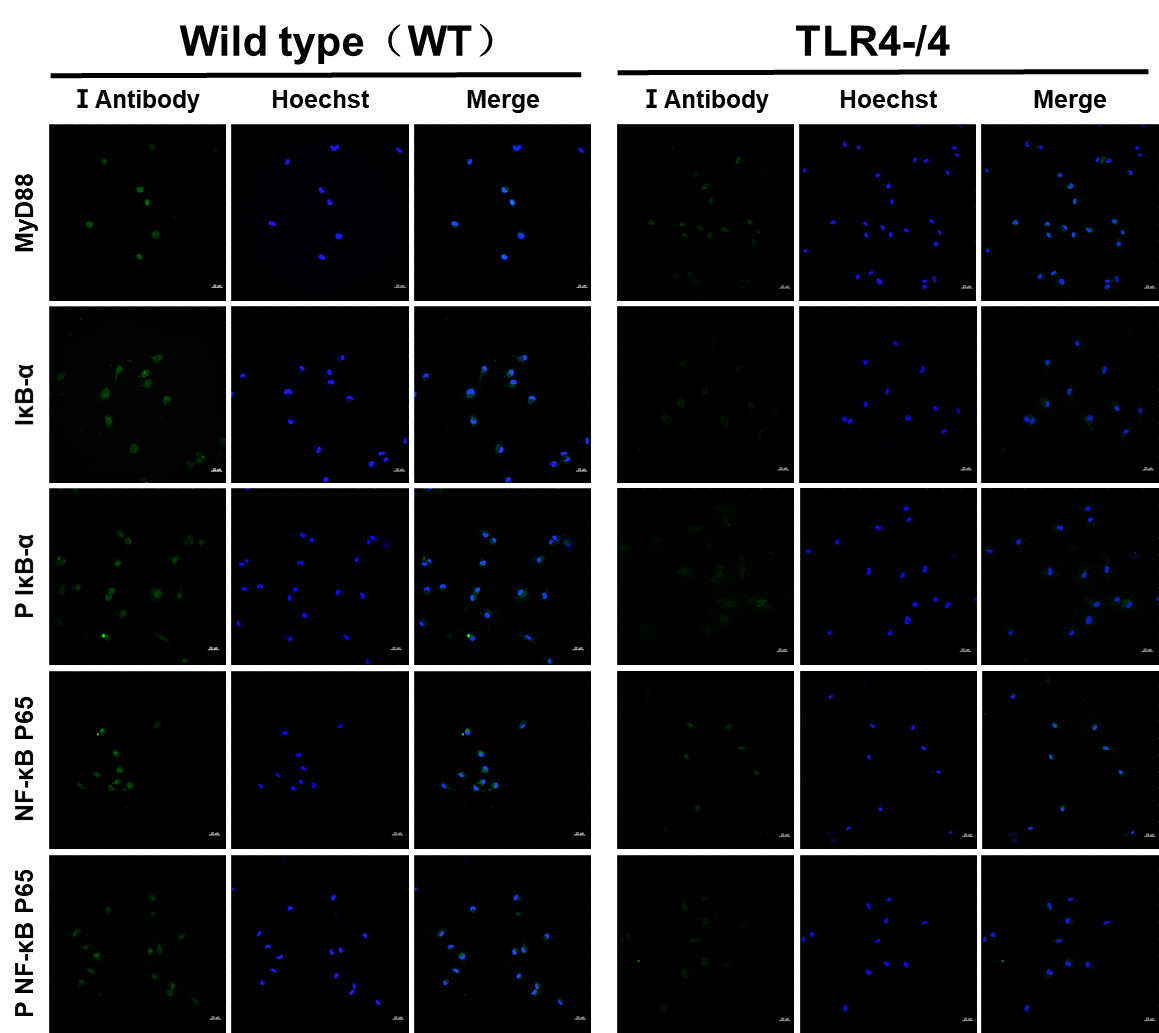


**Figure S5.** The expressions of TLR4, MyD88, IκB-α, P IκB-α, NF-κB P65 and P NF-κB P65 decreased in TLR4-/-Mϕ.


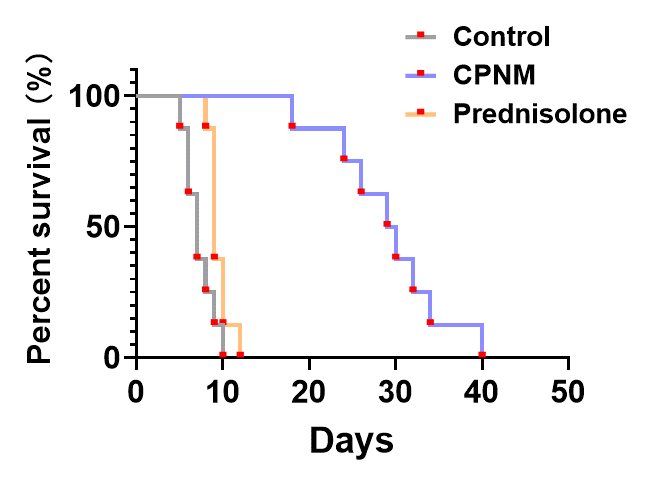
**Figure S6.** Survival curves of rat corneal grafts in each group.

**Table S1.** Clinical Scoring system for the corneal transplantation rejection.

| **Score Clinical Finding** |
| --- |
| Graft opacity  0 Clear cornea  1 Slight haze, details of iris clearly visible  2 Increased haze, some details of iris no longer visible  3 Advanced haze, pupil still recognizable  4 Opaque cornea without view of anterior chamber  Graft edema  0 No edema  1 Mild stromal thickness  2 diffuse stromal edema  3 Pronounced edema with small bleb of epithelium  4 Bullous keratopathy  Neovascularization  0 No neovascularization  1 Neovascularization of peripheral cornea  2 Neovascularization appearing in the graft periphery  3 Neovascularization extending deeper  4 Neovascularization extending to the entire graft |

**Table S2.** Primer used in RT-PCR assay.

| **Gene** | **Primer sequence** |
| --- | --- |
| β-actin | Forwards5’ GAGGGAAATCGTGCGTGAC |
|  | Reverse 5’ CTGGAAGGTGGACAGTGAG |
| IL-6 | Forwards5’ GTGCATCATCGCTGTTCATACAATC |
|  | Reverse 5’ CCACTTCACAAGTCGGAGGCTTA |
| IFN-γ | Forwards5’ AGGCCATCAGCAACAACATAAGTG |
|  | Reverse 5’ GACAGCTTTGTGCTGGATCTGTG |
| IL-1α | Forwards5’ GGGCACAGAGGGAGTCAA |
|  | Reverse 5’ CAGATGGTCAATGGCAGA |
| TNF-α | Forwards5’ TCAGTTCCATGGCCCAGAC |
|  | Reverse 5’ GTTGTCTTTGAGATCCATGCCATT |
| MCP-1 | Forwards5’ CAGCCGACTCATTGGGATCA |
|  | Reverse 5’ CTATGCAGGTCTCTGTCACGCTTC |
| VEGF | Forwards5’ GGCTTTACTGCTGTACCTCC |
|  | Reverse 5’ CAAATGCTTTCTCCGCTCT |

**Table S3.** Template showing the location of cytokine antibodies spotted onto the rat cytokine array c1.


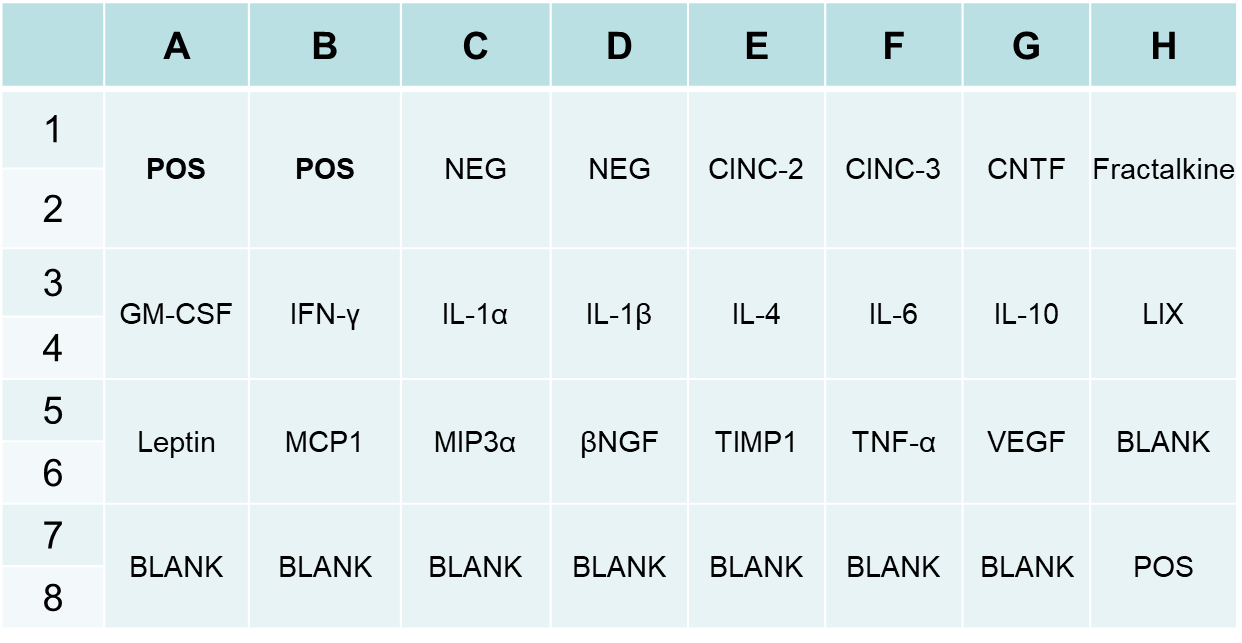


**Table S4.** Template showing the location of cytokine antibodies spotted onto the rat cytokine array c1.


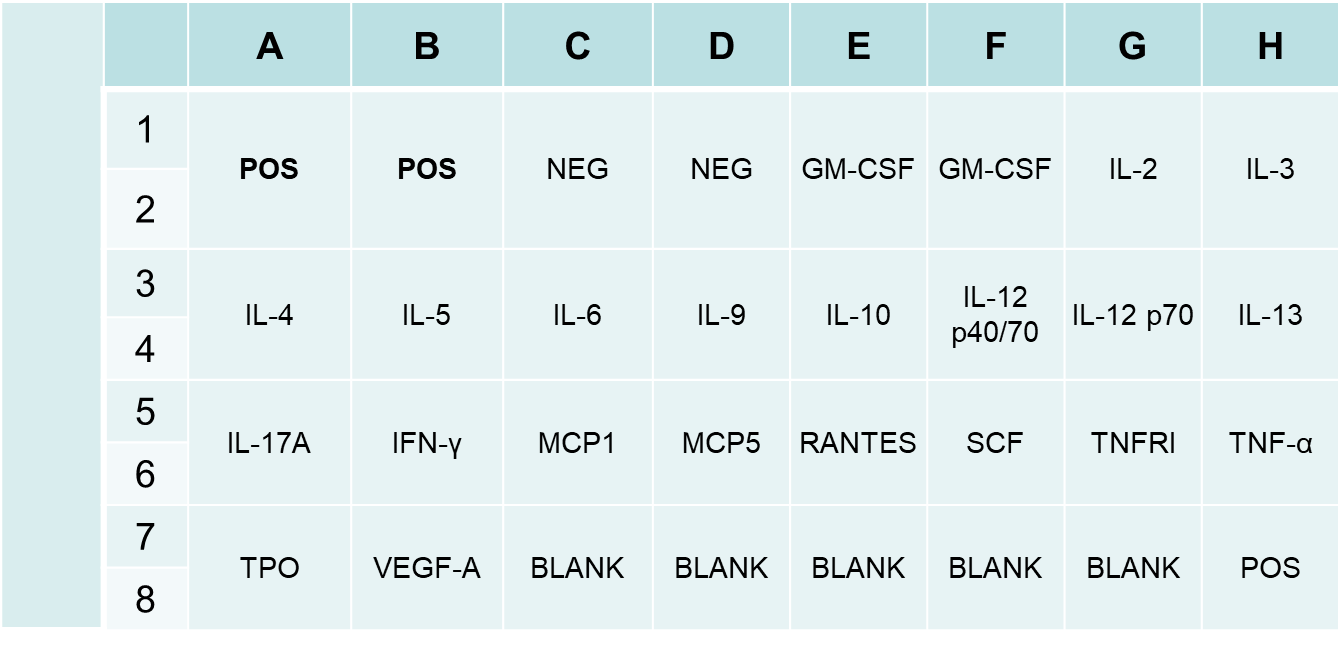

Supplement: Supplementary file 1 — Additional file 1. Figure S1. The particle size of PNMs and NMs. Figure S2. Representative 2PH microscopic 2D (A) and ortho images (B) of corneal in C57BL/6 mice in vivo. Figure S3. The biocompatibility of CPNM. Figure S4. The cytotoxicity of PNM and NM. Figure S5. The expressions of TLR4, MyD88, IκB-α, P IκB-α, NF-κB P65 and P NF-κB P65 decreased in TLR4-/-Mϕ. Figure S6. Survival curves of rat corneal grafts in each group. Table S1. Clinical Scoring system for the corneal transplantation rejection. Table S2. Primer used in RT-PCR assay. Table S3. Template showing the location of cytokine antibodies spotted onto the rat cytokine array c1. Table S4. Template showing the location of cytokine antibodies spotted onto the rat cytokine array c1. [file 12951_2021_1079_MOESM1_ESM.doc]
